# Supplementary material for: Geographical distribution of Enterobacterales with a carbapenemase IMP-6 phenotype and its association with antimicrobial use: An analysis using comprehensive national surveillance data on antimicrobial resistance
Source: PLoS One. 2020 Dec 17;15(12):e0243630. doi: 10.1371/journal.pone.0243630 (PMC7745981; doi:10.1371/journal.pone.0243630)
Supplement: S1 Table — (DOCX) [file pone.0243630.s001.docx]

**S1 Table. The number and proportion of isolates with the IMP-6 phenotype according to specimen types without de-duplication.**

|  | *E. coli* | | *K. pneumoniae* | |
| --- | --- | --- | --- | --- |
|  | 2015 | 2016 | 2015 | 2016 |
| Blood | 21 (7.6%) | 17 (5.6%) | 26 (10.9%) | 27 (10.0%) |
| Respiratory | 53 (19.1%) | 58 (19.0%) | 83 (34.7%) | 78 (28.8%) |
| Urine | 135 (48.6%) | 158 (51.8%) | 62 (25.9%) | 91 (33.6%) |
| Stool | 69 (24.8%) | 72 (23.6%) | 68 (28.5%) | 75 (27.7%) |
| Total | 278 (100%) | 305 (100%) | 239 (100%) | 271 (100%) |
