# Supplementary material for: Geographical distribution of Enterobacterales with a carbapenemase IMP-6 phenotype and its association with antimicrobial use: An analysis using comprehensive national surveillance data on antimicrobial resistance
Source: PLoS One. 2020 Dec 17;15(12):e0243630. doi: 10.1371/journal.pone.0243630 (PMC7745981; doi:10.1371/journal.pone.0243630)
Supplement: S1 Text — (DOCX) [file pone.0243630.s002.docx]

**S1 Text**

A nationwide study in Turkey revealed a negative correlation between ceftriaxone consumption and the prevalence of ceftriaxone resistant *E. coli* and *K. pneumoniae* [34]. Another study pointed out that the emergence and prevalence of antimicrobial resistance cannot solely be explained by a simple selection of antimicrobial therapy [35]. A multivariable analysis of factors of antimicrobial resistance among 103 countries revealed that transmission of antimicrobial resistant bacteria is a dominant contributor to the prevalence of antimicrobial resistance, rather than antimicrobial consumption [36]. Improving sanitation, infection control, clean water, governance, and public expenditure on health-care are all necessary to reduce global antimicrobial resistance. Further studies are warranted to reveal appropriate measures reflecting such factors and to systematically collect and analyze them in addition to the antimicrobial usage data.

**References of S1 Text**

34. Altunsoy A, Aypak C, Azap A, Ergonul O, Balik I. The impact of a nationwide antibiotic restriction program on antibiotic usage and resistance against nosocomial pathogens in Turkey. Int J Med Sci. 2011;8(4): 339-344. Epub 2011/06/08. doi: 10.7150/ijms.8.339. PubMed PMID: 21647326; PubMed Central PMCID: PMCPMC3107466.

35. Merlin C. Reducing the consumption of antibiotics: Would that be enough to slow down the dissemination of resistances in the downstream environment? Front Microbiol. 2020;11: 33-37. Epub 2020/02/13. doi: 10.3389/fmicb.2020.00033. PubMed PMID: 32047488; PubMed Central PMCID: PMCPMC6997526.

36. Collignon P, Beggs JJ, Walsh TR, Gandra S, Laxminarayan R. Anthropological and socioeconomic factors contributing to global antimicrobial resistance: a univariate and multivariable analysis. Lancet Planet Health. 2018;2(9): e398-e405. Epub 2018/09/05. doi: 10.1016/S2542-5196(18)30186-4. PubMed PMID: 30177008.
