# Supplementary material for: Geographical distribution of Enterobacterales with a carbapenemase IMP-6 phenotype and its association with antimicrobial use: An analysis using comprehensive national surveillance data on antimicrobial resistance
Source: PLoS One. 2020 Dec 17;15(12):e0243630. doi: 10.1371/journal.pone.0243630 (PMC7745981; doi:10.1371/journal.pone.0243630)
Supplement: Attachment — Submitted filename: Response to Reviewers.docx [file pone.0243630.s003.docx]

Dear Editor,

We are grateful to the editor and the reviewers for the careful reading of our manuscript. We appreciate the appropriate and constructive comments made, which have helped us to improve our manuscript substantially. We have addressed all reviewer’s comments. Please find enclosed the revised version of the manuscript for your consideration. We have answered all the reviewer’s comments and a separate reply to each of the comments is provided below. The major revisions in the revised manuscript are highlighted in red. The revised tables of the manuscript are attached below.

Editor’s comments:

Thank you for submitting your manuscript to PLOS ONE. After careful
consideration, we feel that it has merit but does not fully meet PLOS
ONE’s publication criteria as it currently stands. Therefore, we invite
you to submit a revised version of the manuscript that addresses the
points raised during the review process.

1. Please ensure that your manuscript meets PLOS ONE's style requirements, including those for file naming. The PLOS ONE style templates can be found at

https://journals.plos.org/plosone/s/file?id=wjVg/PLOSOne_formatting_sample_main_body.pdf and

<https://journals.plos.org/plosone/s/file?id=ba62/PLOSOne_formatting_sample_title_authors_affiliations.pdf>

Response: We have prepared the manuscript in the format required by PLOS One.

2. Please amend the Ethics statement to include the ethics approval information mentioned in the Methods section (i.e., the name of the approval committee and the approval number). Please additionally state whether the need for informed patient consent was waived due to the retrospective nature of the study and the use of de-identified data. Additionally, while we note that you have provided your data via a link to Github, we also ask that you provide any relevant code (i.e., the 'in-house' Perl script mentioned in the manuscript).

Response: We wrote the approval for extraction and use of the data in the Methods section as follows: “Approval for extraction and use of the data was granted by the Ministry of Health, Labour and Welfare (Approval no. 1010-5).”.

Response: We have provided the in-house Perl script in the GitHub page below. <https://github.com/bioprojects/IMP-6_phenotype_distribution>

3. We note that [Figure(s) 1] in your submission contain [map/satellite] images which may be copyrighted. All PLOS content is published under the Creative Commons Attribution License (CC BY 4.0), which means that the manuscript, images, and Supporting Information files will be freely available online, and any third party is permitted to access, download, copy, distribute, and use these materials in any way, even commercially, with proper attribution. For these reasons, we cannot publish previously copyrighted maps or satellite images created using proprietary data, such as Google software (Google Maps, Street View, and Earth). For more information, see our copyright guidelines: http://journals.plos.org/plosone/s/licenses-and-copyright.

 We require you to either (1) present written permission from the copyright holder to publish these figures specifically under the CC BY 4.0 license, or (2) remove the figures from your submission:

1.    You may seek permission from the original copyright holder of Figure(s) [#] to publish the content specifically under the CC BY 4.0 license.

We recommend that you contact the original copyright holder with the Content Permission Form (http://journals.plos.org/plosone/s/file?id=7c09/content-permission-form.pdf) and the following text:

“I request permission for the open-access journal PLOS ONE to publish XXX under the Creative Commons Attribution License (CCAL) CC BY 4.0 (http://creativecommons.org/licenses/by/4.0/). Please be aware that this license allows unrestricted use and distribution, even commercially, by third parties. Please reply and provide explicit written permission to publish XXX under a CC BY license and complete the attached form.”

Please upload the completed Content Permission Form or other proof of granted permissions as an "Other" file with your submission.

In the figure caption of the copyrighted figure, please include the following text: “Reprinted from [ref] under a CC BY license, with permission from [name of publisher], original copyright [original copyright year].”

2.    If you are unable to obtain permission from the original copyright holder to publish these figures under the CC BY 4.0 license or if the copyright holder’s requirements are incompatible with the CC BY 4.0 license, please either i) remove the figure or ii) supply a replacement figure that complies with the CC BY 4.0 license. Please check copyright information on all replacement figures and update the figure caption with source information. If applicable, please specify in the figure caption text when a figure is similar but not identical to the original image and is therefore for illustrative purposes only.

The following resources for replacing copyrighted map figures may be helpful:

USGS National Map Viewer (public domain): http://viewer.nationalmap.gov/viewer/

The Gateway to Astronaut Photography of Earth (public domain): http://eol.jsc.nasa.gov/sseop/clickmap/

Maps at the CIA (public domain): https://www.cia.gov/library/publications/the-world-factbook/index.html and https://www.cia.gov/library/publications/cia-maps-publications/index.html

NASA Earth Observatory (public domain): http://earthobservatory.nasa.gov/

Landsat: http://landsat.visibleearth.nasa.gov/

USGS EROS (Earth Resources Observatory and Science (EROS) Center) (public domain): http://eros.usgs.gov/#

Natural Earth (public domain): http://www.naturalearthdata.com/

Response: The map was created using a publicly available file of Natural Earth
(<https://www.naturalearthdata.com/about/terms-of-use/>). As written in the Terms of Use, it is in the public domain and does not require any permission to use it in any manner.

Reviewers' comments:

Reviewer #1: I would like to thank the editors for sending me such an
interesting and relevant manuscript to review. I believe that the
findings from this study are very important and have the ability not
only to inform antimicrobial stewardship, but provide a good example of
for other countries to investigate their own antimicrobial resistance
and usage relationships.

I have a few comments for the authors, which I hope are of use.

General
- Would the authors consider using more current taxonomy i.e. Enterobacterales as opposed to Enterobacteriaceae [https://doi.org/10.1099/ijsem.0.001485]?

Response: We have amended to use “Enterobacterales” instead of “Enterobacteriaceae” throughout the revised manuscript accordingly.

- Use a consistent approach to reporting p-values e.g. 1 significant figure, 2 decimal places etc.

Response: We have amended for the p-values aligned to three decimal places and for the percentages aligned to one decimal place in the revised manuscript, accordingly.

Line 38: Suggest remove “This examination revealed a skewed frequency distribution.” This sentence means nothing in isolation.

Response: We removed the sentence in the revised manuscript accordingly.

Line 39-40: Were high rates observed in prefectures with high levels of 3GC prescribing? If so, suggest this sentence is merged with following sentence.

Response: We merged the sentence to the following sentence as follows: “Isolates were detected in approximately half of all prefectures, and combined analysis with the national data of antimicrobial usage revealed a statistically significant association between the frequency and usage of not carbapenems but third-generation cephalosporins (*p* = 0.006, logistic mixed-effect regression) and a weaker association between the frequency and usage of fluoroquinolones (*p* = 0.043).” in line 35.

Line 44 and throughout: Remove “not carbapenems”. I don’t think you need to precede your 3GC observations with “not carbapenems” after the first mention.

Response: We removed “not carbapenems” in the revised manuscript, accordingly.

Abstract: Could you conclude the abstract with a public health/antimicrobial stewardship recommendation? I believe the findings are important and actionable.

Response: We have concluded the abstract as follows: “We expect the findings will promote antimicrobial stewardship to reduce the spread of the notable carbapenemase gene.” in line 41-43.

Line 53: Change “species” to “genera”

Response: We changed “species” to “genera” in the revised manuscript, accordingly.

Line 63-65: This is a repeated statement – you have already stated that IMP carbapenemases are plasmid-mediated etc. earlier in the Introduction.

Response: We removed the sentence in the revised manuscript, accordingly.

Lines 80-87: Summarise this section further. Suggest remove sentences between line 81 and 85 as you have already referenced the study and therefore detailed findings do not need to be provided.

Response: We summarized this part as follows: “In 2018, 2289 CRE cases were reported and 1684 CRE isolates were analyzed, which revealed that the *bla*_IMP_ gene was the most common (254, 85.5%) out of the 297 isolates positive for carbapenemase genes, and *bla*_IMP-6_ and *bla*_IMP-1_ are two dominant genes in CRE in Japan (52.0% and 46.3% among 123 genome-sequenced isolates with *bla*_IMP_ genes). The incidence of infections caused by CRE with *bla*_IMP-6_ varied by region in the report” in the revised manuscript in lines 76-81.

Lines 92-96. This statement would be better placed in the Discussion, to highlight the advantages of the study design.

Response: We moved this statement to the first paragraph of the Discussion as follows: “Such a utilization of the phenotypic data of bacterial culture and antimicrobial susceptibility testing that are routinely conducted in hospitals throughout a country is complementary and cost-effective compared to genetic testing by sequencing to detect *bla*_IMP-6_, which requires additional time and cost and currently cannot be extended to potential asymptomatic carriers across Japan.” in lines 217-221, accordingly. In addition, we added the sentence “The data include results of bacterial culture and antimicrobial susceptibility testing that are routinely conducted in the hospitals.” to the Introduction in lines 89-91.

Lines 104-111: This reads more like an introduction of the methods. Suggest ending the Introduction by stating the aim(s) of your study.

Response: , We have amended the section in the revised manuscript as follows: “In this study, we aim to determine the geographical distribution of *E. coli* and *K. pneumoniae* isolates showing a pattern indicative of the IMP-6 phenotype (imipenem susceptible, meropenem resistant), using the JANIS database since the susceptibility pattern of *bla*_IMP-6_-positive strains is unique.” in lines 96-99, accordingly.

Line 125: Defined daily dose = DDD

Response: We have amended the definition of DID in line 118, accordingly.

Line 160: Change “species” to “genera”

Response: We changed “species” to “genera” in the revised manuscript, accordingly.

Lines 163-166: The detail in this sentence is superfluous since you have described the dataset and study years in the Methods. Suggest the sentence is simplified.

Response: We simplified the sentence in the revised manuscript as follows: “Geographical distribution of frequency of *E. coli* and *K. pneumoniae* isolates with the IMP-6 phenotype is shown as prefectural maps in Fig 1.” in lines 155-156, accordingly.

Line 168: Add “respectively” after “2016”.

Response: We amended the sentence as follows: “The percentages of prefectures where the isolate with IMP-6 phenotype is undetectable were 42.6% and 55.3% for *E. coli* in 2015 and 2016, and 42.6% for *K. pneumoniae* in both 2015 and 2016, respectively.” in the revised manuscript in lines 158-160.

Line 178: Provide the actual number (since this is the frequency) with the proportion in brackets immediately afterwards.

Response: We added the actual number in the revised manuscript as follows: “The highest frequency was 1.7% (28/1644) for *E. coli* and 1.6% (13/790) for *K. pneumoniae*.” in lines 170-171, accordingly.

Line 179-180: This needs to precede the reporting of frequency and associated proportions. Also need to clarify what this “average” represents, and the type of “average” reported i.e. mean, median, mode?

Response: We moved the sentence and changed the word from “average” to “mean” in the revised manuscript as follows: “The mean of total number of antimicrobial susceptibility tests as denominator of the frequency was 4241 for *E. coli* and 2185 for *K. pneumoniae*.” in lines 168-169.

Line 185: Change “average” to “median”.

Response: We avoided to use “average” and changed to “mean” in the revised manuscript. In the figure, the middle of the diamond indicates the mean, whereas the vertical line in the box indicates the median.

Lines 191-192: Report 2015 data before 2016 (as you have done for the rest of the manuscript).

Response: We have amended the order in the revised manuscript as follows: “The total number of *E. coli* isolates with the IMP-6 phenotype after de-duplication was 221 in 2015 and 242 in 2016. The corresponding numbers for *K. pneumoniae* were 189 in 2015 and 201 in 2016.” in lines 181-183.

Lines 199-200: It is not clear which p-value relates to which specimen type.

Response: We have amended the order in the revised manuscript as follows: “No statistically significant difference in the proportion among the four specimen types was found between the two conditions (with and without de-duplication) in 2015 and 2016 for *E. coli* (*p* = 0.204 and *p* = 0.795, respectively), and for *K. pneumoniae* (*p* = 0.862 and *p* = 0.478, respectively) by chi-square test.” in lines 188-191.

Lines 206-210: This statement is more suited to the Discussion.

Response: We moved the statement to the second paragraph of the Discussion in the revised manuscript in lines 223-229, accordingly.

Lines 211-215: Much of this is already covered in the Methods and can be removed.

Response: ,We simplified the explanation of Fig 3 in the revised manuscript as follows: “The relationship between the frequency and antibiotic usage (DID) (Fig 3) clearly revealed almost no relationship between the usage of carbapenems and the frequency of the IMP-6 phenotype,” in lines 192-194.

Lines 215-218: The wording of this goes beyond reporting the results, introducing elements of interpretation, which belongs in the Discussion.

Response: We removed the words “the unexpected finding of” and amended as shown above in lines 192-194 in the revised manuscript.

Lines 241-242: If the proportion of Enterobacter cloacae is so high, why were these isolates not included in the study? An explanation in the Introduction or Methods (depending on reason) may be necessary.

Response: The isolates of *Enterobacter cloacae* harboring *bla*_IMP-1_ are frequently reported, but it is not the case for *bla*_IMP-6_. The most frequently reported bacteria of the isolates with *bla*_IMP-6_ are *E. coli* and *K. pneumoniae*. We have removed the Lines 241-242 that didn’t distinguish the two alleles, and instead added the sentence “The most frequently reported bacterial species harboring *bla*_IMP-6_ are *E. coli* and *K. pneumoniae*, although the number of publications is small” to Introduction (lines 72-73).

Lines 242-245: This information belongs in the Results.

Response: The sentences did not describe the results of our study, but those in a previous report. We replaced the sentences with “The results are consistent with the previous report of NESID that the isolates with *bla*_IMP-6_ are detected only in some prefectures” in lines 228-229.

Lines 262-265: This information belongs in the Results.

Response: We moved the information to the end of Results as follows: “In the isolates with the IMP-6 phenotype, resistance rate of ceftazidime, as a representative third-generation cephalosporins, was 94.4% of the *E. coli* and 93.0% of the *K. pneumoniae* isolates across the prefectures.” in lines 201-204.

Lines 269-271: Is there a reference for this?

Response: We apologize for the confusion. The sentence mentioned the result of our study. We have removed “as prior data from our group has demonstrated”, and modified the sentence as follows: “The remaining 5.6% of *E. coli* and 7.0% of *K. pneumoniae* isolates examined in our study are unlikely to carry *bla*_IMP-6_, contrary to the phenotype.” in lines 244-246.

Figure 1: The category “<1” does not make sense in the legend.

Response: We changed the name of the category to “≥ 1%” which means higher than 1% in the Fig 1.

Figure 3: I think this data would be better presented as a table.

Response: We changed the Fig 3 and S1 Fig to tables, accordingly as below.

**Table 1.**

**The number and proportion of isolates with the IMP-6 phenotype according to specimen types after de-duplication.**

|  | *E. coli* | | *K. pneumoniae* | |
| --- | --- | --- | --- | --- |
|  | 2015 | 2016 | 2015 | 2016 |
| Blood | 7 (3.2%) | 9 (3.7%) | 17 (9.0%) | 12 (6.0%) |
| Respiratory | 46 (20.8%) | 47 (19.4%) | 72 (38.1%) | 59 (29.4%) |
| Urine | 109 (49.3%) | 128 (52.9%) | 48 (25.4%) | 70 (34.8%) |
| Stool | 59 (26.7%) | 58 (24.0%) | 52 (27.5%) | 60 (29.9%) |
| Total | 221 (100%) | 242 (100%) | 189 (100%) | 201 (100%) |

**S1 Table**

**The number and proportion of isolates with the IMP-6 phenotype according to specimen types without de-duplication.**

|  | *E. coli* | | *K. pneumoniae* | |
| --- | --- | --- | --- | --- |
|  | 2015 | 2016 | 2015 | 2016 |
| Blood | 21 (7.6%) | 17 (5.6%) | 26 (10.9%) | 27 (10.0%) |
| Respiratory | 53 (19.1%) | 58 (19.0%) | 83 (34.7%) | 78 (28.8%) |
| Urine | 135 (48.6%) | 158 (51.8%) | 62 (25.9%) | 91 (33.6%) |
| Stool | 69 (24.8%) | 72 (23.6%) | 68 (28.5%) | 75 (27.7%) |
| Total | 278 (100%) | 305 (100%) | 239 (100%) | 271 (100%) |
